# Quality of life in patients in South Korea requiring special care after fixed implants: a retrospective analysis

**DOI:** 10.1186/s12903-023-03753-x

**Published:** 2023-12-14

**Authors:** Soo-Yeon Yoo, Hyun-Jong Kim, Seong-Kyun Kim, Seong-Joo Heo, Jai-Young Koak, Ji-Man Park

**Affiliations:** 1 Department of Prosthodontics & Dental Research Institute, School of Dentistry, Seoul National University Dental Hospital, Seoul National University, 101 Daehak-ro, Jongno-gu, Seoul, 03080 Korea; 2 Private Practice, Seoul, Korea

**Keywords:** Oral health (OH), Quality of life (QoL), Impact Profile (IP), Disabled, Persons with disabilities, Dental implants, Prostheses and implants

## Abstract

**Background:**

The study on oral health-related quality of life (OHRQoL) of disabled patients is rare but critical for welfare of patients. The aim of this study was to examine the effect of fixed implants in edentulous areas on OHRQoL in Korean disabled patients.

**Methods:**

The OHRQoL of 63 disabled individuals was evaluated using the Oral Health Impact Profile (OHIP)-14 questionnaires and studied by potential affecting variables such as age, sex, disability severity, and time of disability acquisition. Wilcoxon-signed rank tests were used to examine the OHIP-14 scores for those who had pre/post-fixed implants. Multiple linear regression analysis was used to examine the relationships between factors and OHIP-14 scores before and after implants. A partial correlation analysis was also performed to determine which variables influenced OHIP-14 scores before and after treatment. The Mann-Whitney test was employed for sex and time of disability acquisition analysis (α = 0.05).

**Results:**

Significant improvement was found in OHIP-14 post-implant treatment scores *(P* < .001). After implant treatment, the severity of disability produced significantly different results (*P* = .009). Pearson’s correlation coefficient between severity of disability and pre/post-implant OHIP-14 scores was 0.265 (*P* = .030). After controlling for severity of disability, the results showed older patients had lower OHIP-14 scores (*P* = .032). No differences were found for sex or time of disability acquisition (congenital vs. acquired).

**Conclusions:**

Fixed implant treatment improved OHRQoL for disabled patients, and the severity of disability was positively correlated with improvement of OHRQoL. For patients with a similar level of disability, the OHRQoL decreased with age.

## Background

In 2001, the World Health Organization (WHO) comprehensively defined disability as a disorder in body structure and function based on a medical model [1]. A key point in the definition is that healthcare/dental practitioners should consider not only the medical concept of disability but also the public concept of quality of life (QoL) in people with special needs. In many countries around the world, the concept of disability has been transformed into a path toward improving one’s quality of life [2].

In Korea, there are two subsets of disability: mental disability including developmental disabilities such as mental retardation/autism and mental disorders, and physical disabilities including visceral disorders such as liver and kidney disorders and physical impairments such as communication/facial disorders. Previously, disabled patients in Korea were categorized according to the severity of their disability using a 6-point grade, from 1 to 6; since 2019, this grading was further adjusted, with 1–3 grades called ‘severe’ and 4–6 grades categorized as ‘mild’. Severe mental/physical impairment results in activity limitation and social restriction. Isolation and loss of socio-economic activity reduce QoL. Therefore, to improve QoL of disabled patients, healthcare/dental practitioners aim to address the primary needs of patients in their respective fields. In dentistry, to maintain nutrition, clinicians should prioritize the restoration of normal oral function, including mastication and swallowing.

Due to cognitive deficiencies, lack of dexterity, and muscle function issues, disabled people frequently struggle to maintain their dental hygiene. As a result, the dental health of individuals with disabilities is frequently jeopardized [3, 4]. Disabled patients are more likely to have missing teeth, caries, and periodontitis, and only a small percentage of patients receive dental care [3, 4]. Furthermore, those with disabilities may confront difficulties such as involuntary lower jaw movement and unstable occlusion, poor cooperation, and highly tensed muscles that restrict dental treatment. As a result, from a clinician’s perspective, patients with disabilities appear to have lower OHRQoL than those without disabilities. Improving OHRQoL by restoring missing teeth in disabled patients is challenging but worthwhile.

In previous studies, fixed implants or removable prostheses using implants were shown to improve OHRQoL of partially or fully edentulous patients [5, 6]. Implant studies on disabled patients are rare; the prognosis of implants in intellectually disabled patients and meta-analysis of implant-based treatment in immunocompromised patients were all published implant studies on disabled patients [7, 8]. As a result, OHRQoL of disabled individuals following fixed implant treatments is rarely studied but this type of research is required to consider welfare improvement of disabled patients.

OHRQoL may be strongly influenced by psychosocial behavior and subjective feelings of security, making it difficult to set appropriate OHRQoL expectations. The Oral Health Impact Profile (OHIP)-49 questionnaires developed by Loker [6] have been used as subjective tools to evaluate OHRQoL for decades. The OHIP-14 is a widely used short version of the OHIP-49 with validated performance reliability that consists of questions about oral health [9–12].

The aim of this study was to verify changes in OHRQoL following fixed implant treatments in patients with special needs. The OHRQoL of disabled patients as measured by OHIP-14 questionnaires was investigated and analyzed in relation to potential confounding variables such as age, sex, disability severity, and time of disability acquisition. The null hypothesis for this study was that OHRQoL is unaffected by the severity of disabilities.

## Methods

Our study sample was drawn from 93 patients with special needs who were treated with fixed implants at Seoul National University Dental Hospital and Seocho-Gu Public Health Center in South Korea between January 2004 and December 2020. This study was authorized by the Institutional Review Board of Seoul National University School of Dentistry (No. S-D20210007). All patients included in this study were treated by surgical or prosthodontic specialists and underwent periodic recall check. Patients who were unable to understand questions or communicate with others were excluded, and the OHRQoL of the final 63 participants was examined after implant treatment using OHIP-14 questionnaires. The 63 disabled patients and their caregivers were asked to rate experiences with dental problems and satisfaction at least 6 months after receiving fixed implant prostheses. Disability severity was followed by disability grade classification in accordance with Korean Ministry of Health and Welfare (KMHW) regulations. The severity of disability was calculated, and all disabled subjects were classified as medical status based on their received grade (1–6) according to the medical guideline of KMHW. Furthermore, the severity of disability defined in this study reflected the difficulty that both clinicians and patients may encounter during dental treatment. For the analysis of this study, the lowest value of 7 was given to four people who had disabilities irrelevant to oral health because their disabilities had nothing to do with dental treatment. Two of the four patients had knee bone problems and two had one kidney; all live regular lives except for their discomfort and periodic medical check-ups.

The OHIP-14 includes 14 questions scored using 5-point scales, for a total score of 70 (Table 1). Subjective ability was evaluated using a 5-point Likert scale, with 1 indicating “it is frequently very difficult and inconvenient” and 5 indicating “it is satisfactory and not at all inconvenient.” A higher OHIP-14 score indicates better OHRQoL.

**Table 1:** Questions analyzed in this study based on OHIP-14

Pre-treatment	Post-treatment
1. Have you ever felt uncomfortable with your pronunciation? (pronunciation/speaking)	1. Have you ever felt uncomfortable with your pronunciation? (pronunciation/speaking)
2. Have you ever felt that your sense of taste is decreased? (taste)	2. Have you ever felt that your sense of taste is worse than before? (taste)
3. Have you ever had pain in your tongue, under your tongue, or on the roof of your mouth? (mastication and oral pain/comfort)	3. Have you ever had pain in your tongue, under your tongue, or on the roof of your mouth? (mastication and oral pain/comfort)
4. Have you ever had trouble eating? (mastication/swallowing)?	4. Have you ever had trouble eating? (mastication/swallowing)?
5. Have you ever been reluctant to meet other people because of embarrassment? (esthetics)	5. Have you ever been reluctant to meet other people because of embarrassment? (esthetics)
6. Have you ever been very nervous (about your appearance)? (esthetics)	6. Have you ever been very nervous (about your appearance)? (esthetics)
7. Have you ever been unhappy with your diet? (mastication/swallowing)	7. Have you ever been unhappy with your diet? (mastication/swallowing)
8. Have your ever stopped eating in the middle of a meal? (mastication and oral pain)	8. Have your ever stopped eating in the middle of a meal? (mastication and oral pain)
9. Have you ever been unable to rest comfortably? (general condition – physical function)	9. Have you ever been unable to rest comfortably? (general condition – physical function)
10. Have you ever felt embarrassed by your eating?	10. Have you ever felt embarrassed by your eating?
11. Have you ever been tempted to get angry with other people? (general - psychological)	11. Have you ever been tempted to get angry with other people? (general - psychological)
12. Have you ever found it difficult to do what you normally do? (general - physical function)	12. Have you ever found it difficult to do what you normally do? (general - physical function)
13. Have you ever felt that life is less satisfying than it used to be? (general - psychological)	13. Have you ever felt that life is less satisfying than it used to be? (general - psychological)
14. Have you ever been unable to do your part at all, mentally, physically and socially? (general -physical. Psychological)	14. Have you ever been unable to do your part at all, mentally, physically and socially? (general -physical. Psychological)

Subjects (with help of caregivers) also answered questions about improvement of masticatory/swallowing function and esthetic appearance using a visual-analog-scale (VAS). Improvements following treatments were rated using a 10-point VAS ranging from “hardly ever”=1 to “very much”=10. A higher VAS score indicates greater function and esthetics.

All data were evaluated using SPSS version 25 (SPSS Inc.). Cronbach’s α was assessed to verify the reliability of OHIP-14 questions. Statistical analyses were performed for all variables. OHIP-14 scores at pre/post-fixed implant times were analyzed with the Wilcoxon-signed rank test. Associations between variables (age, severity of disabilities (1–7 grades)) and OHIP-14 scores at pre/post-fixed implant treatment were analyzed using multiple linear regression analysis. The significance level was set to 0.05. A partial correlation analysis was performed to clarify which variables affected OHIP-14 scores pre/post-treatment. The Mann-Whitney test was used for analysis according to sex and time of disability acquisition (congenital vs. acquired). Functional and esthetic improvement after treatment was analyzed by Wilcoxon-signed ranked test.

Overall oral and general condition-related QoL scores using each subscale from the study of Sonoyama et al. [5] were also calculated. The subscale scores were normalized by dividing the total score by the subscale’s possible maximum value and expressed as a percentage. The mean differences in overall OHRQoL scores were analyzed using the Wilcoxon-signed rank test. Significance was set at the 0.05 level.

## Results

**Fig. 1:**
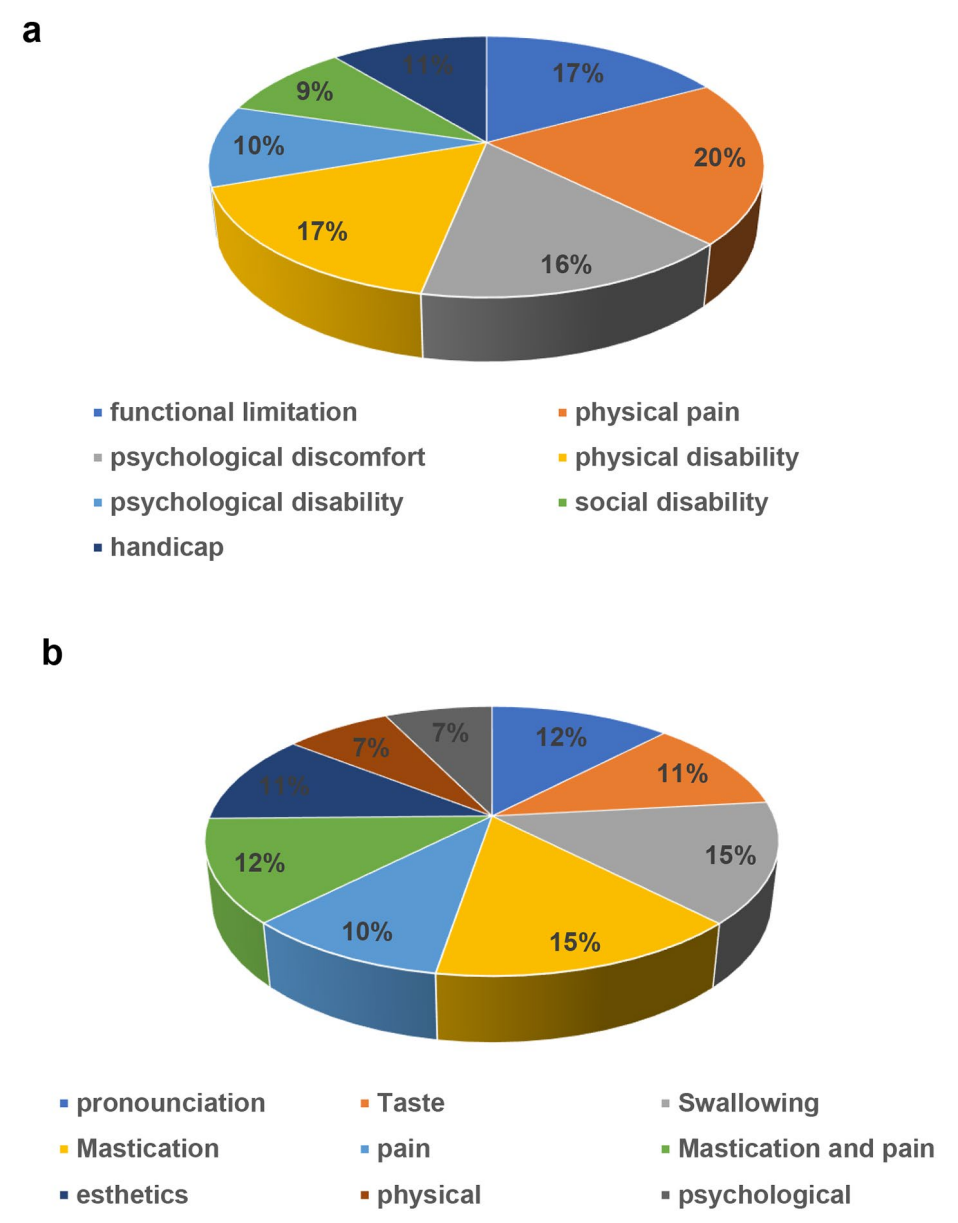
%differences in OHRQoL improvement before and after implant treatments. **a** OHIP-14 questionnaires revealed %differences in OHRQoL scores between before and post implant treatments. **b** Pre/post treatment %differences in oral- and general condition-related QoL ratings

This study included 63 participants with a mean age 63.16 ± 11.57 years. Table 2 shows the characteristics of subjects. Cronbach’s α was 0.94 pre-treatment and 0.93 post-treatment. Therefore, the reliability of OHIP-14 questions in this study was verified. The average time from implant loading to answering the OHIP-14 questions was 24.71 months. Significant improvements from mean OHIP-14 baseline to post-implant treatment scores were found in all subscales including functional limitations, physical pain, psychological discomfort, physical disability, social disability, and handicap (Table 3; Fig. 1). OHIP-14 domains 3 and 4, which deal with physical pain, improved the most (% difference = 35.26), while OHIP-14 domains 11 and 12, which deal with social disability, improved the least (% difference = 16.33).

**Table 2:** Characteristics of the study subjects

Variables	n	Note			
Age (years)	63	Range: 30–78			
Sex					
Male	24				
Female	39				
Severity of disability					
Severe brain lesion/epilepsy	17	High-grade disability			
Severe mental disability	0	High-grade (1–3) autism, intellectual or psychiatric disorder			
Severe physical disability	26	High-grade (1–3) physical disorder			
Mild mental disability	0	Low-grade (4–6) autism, intellectual or psychiatric disorder			
Mild physical disability	16	Low-grade (4–6) physical disorder			
Disability irrelevant to oral health	4	Hearing disorder, visual impairment Low grade (4–6) physical disability; a value of 7 was used for calculation in this study			
Timing of disability acquisition					
Congenital	51				
Acquired	12	In an accident			

**Table 3:** OHRQoL scores of pre/post implant treatment

OHIP-14 Domain	n	Pre		Post		*P*	% Difference
		Mean	±SD	Mean	±SD		
Functional limitation (1,2)	126	3.23	1.29	4.58	0.81	< 0.001	29.58
Physical pain (3,4)	126	2.92	1.43	4.52	0.89	0.001	35.26
Psychological discomfort (5,6)	126	3.42	1.37	4.7	0.69	0.007	27.31
Physical disability (7,8)	126	3.34	1.42	4.68	0.64	0.004	28.47
Psychological disability (9,10)	126	3.96	1.15	4.8	0.04	0.046	17.49
Social disability (11,12)	126	4.06	1.25	4.85	0.48	0.002	16.33
Handicap (13,14)	126	3.76	1.39	4.65	0.07	< 0.001	18.94

Abbreviations: OHRQoL, oral health related quality of life; OHIP, oral health impact profile; SD, standard deviation

OHIP-14 domains were also analyzed following oral and general condition-related QoL according to the classification of Sonoyama et al. [5] (Table 4; Fig. 1). There were significant differences in QoL scores for oral and general conditions. The mastication function in oral function improved the most (% difference = 35.40). General condition-related QoL was lower than oral condition-related QoL.

**Table 4:** Oral- and general condition–related QoL scores of pre/post implant treatment

Domain (OHIP-14)	n	Pre (%)		Post (%)		*P*	% Difference
		Mean	±SD	Mean	±SD		
Oral functions							
Pronunciation (1)	63	63.17	28.33	92.06	15.46	< 0.001	28.89
Taste (2)	63	65.07	24.87	91.42	16.74	0.035	26.35
Swallowing (7)	63	58.41	28.12	92.69	13.58	0.08	34.28
Mastication (4,7)	126	55.39	27.84	90.79	16.71	0.014	35.4
Pain (3,8)	126	70.16	28.14	93.33	14.31	< 0.001	23.17
Mastication and pain (3,4,7,8)	252	62.77	28.91	92.06	15.58	< 0.001	29.29
Appearance							
Esthetics (5,6)	126	67.46	28.81	93.34	16.19	0.006	25.88
General							
Physical (9,12)	126	79.2	24.44	96.19	11.23	0.002	16.99
Psychological (10,11,13,14)	189	78.3	25.52	95.49	13.11	< 0.001	17.19

Abbreviations: QoL, Quality of life; OHIP, oral health impact profile; SD, standard deviation

Disability severity was associated with significantly different results in OHIP-14 of pre- and post-treatment (*P* = .009) according to multiple linear regression. Pearson’s correlation coefficient for the relationships between severity of disability and differences in pre/post-treatment OHIP-14 scores was 0.265 (*P* = .030). After the severity of disability was controlled, the results showed that older subjects had lower OHIP-14 scores. The partial correlation coefficient r of age was − 0.274 (*P* = .032). OHIP-14 scores did not differ pre/post-treatments for sex or time of disability acquisition (congenital vs. acquired).

**Fig. 2:**
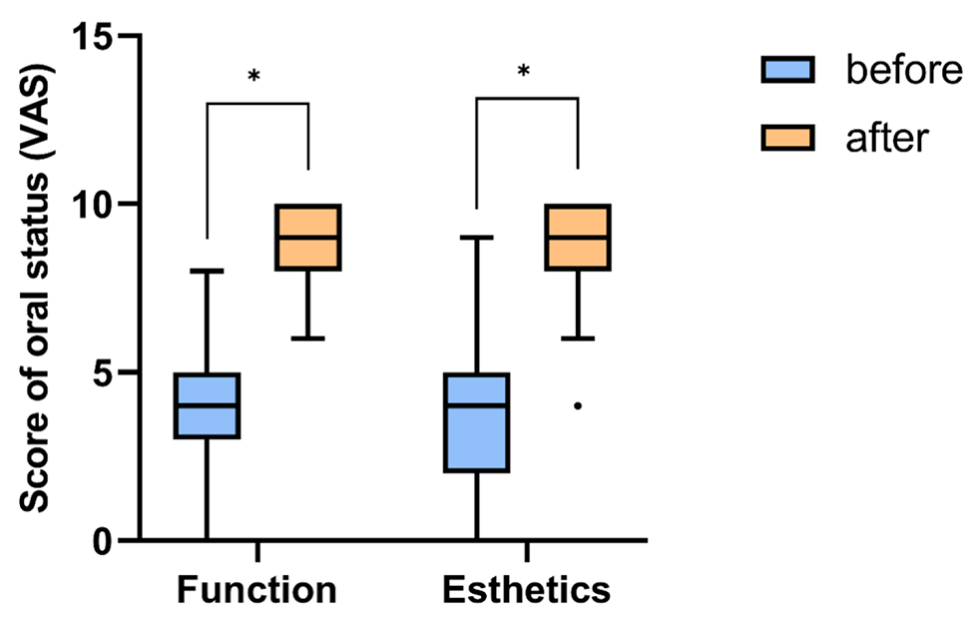
Oral status scores. Blue bars indicate the scores of functional and esthetics ratings before fixed implant treatments in disabled patients, and yellow bars indicate the scores of functional and esthetics ratings after fixed implant treatments. There were statistically significant increases of ratings after treatment. * means *P* < .001

There were also significant increases in VAS value in functional and esthetic improvement after fixed implant treatment compared with before treatment (*P* < .001, <0.001, respectively). The mean differences of improvement were 5.06 ± 2.16 in function and 4.63 ± 2.37 in esthetics (Fig. 2). No implants failed in disabled patients (Table 5).

**Table 5:** Complications of fixed implants in the study group

Complications	Proportion (%)
Implant failure	0
Dislodgement of prosthesis	3.17
Porcelain fracture	1.5
Contact loosening	4.7

## Discussion

The number of disabled people is increasing because of broadened concepts of disability as well as the development of medical examination technology. However, dental treatments for disabled patients remain challenging and have been neglected due to the complicated medical situations of disabled patients. Dental implants have been developed and used for several decades. Dental implants have been shown to positively affect partially/fully edentulous patients and improve their OHRQoL [13]. It is time to consider how fixed dental implants can improve the OHRQoL of disabled individuals. To the best of our knowledge, study on OHRQoL of dental implant treatments for disabled patients is rare.

More time and effort should be applied to implant treatment of disabled individuals because of limitations including spasticity, uncontrolled physical condition, lack of understanding, and other factors. The cost of implants is often prohibitive because disabled patients are generally poorer than the non-disabled [14, 15]. Therefore, fixed implant treatment can be a greater financial burden to disabled individuals compared with non-disabled individuals. Due to lack of cost-effectiveness, it was assumed that the improvement of OHRQoL might be decreased in disabled patients compared with non-disabled patients. Furthermore, patients with disabilities in the current study were advised of an unexpectedly bad prognosis before implant treatment, according to the clinical chart. As a result, fixed implants that place a financial burden on disabled patients may be risky procedures that do not ensure a safe prognosis. Awad et al. found that OHRQoL (assessed by OHIP-49) was significantly increased in non-disabled patients treated with dental implants [16]. Our results are consistent with these previous results of the non-disabled. The OHIP-14 scores of disabled patients were significantly improved after implant treatment (*P* < .001). Questions concerning psychological discomfort and psychological disability (among OHIP-14) showed greater improvement in disabled patients.

However, compared with Sonoyama et al.’s study, [4] disabled patients were likely to show better satisfaction than non-disabled patients. Sonoyama et al. [4] used self-administered QoL questionnaires with two major subscales, oral- and general condition–related QoL. Therefore, we applied a similar classification to OHIP-14 domains to analyze the oral and general condition-related QoL of disabled individuals. In Sonoyama et al., the mean scores of mastication/comfort, pronunciation, esthetics, physical function, and psychological state in non-disabled patients were 86.9, 94.5, 88.5, 71.7, and 78.8, respectively, following fixed implant treatments. In contrast, in our study, all mean scores of those questions following fixed implant treatments were greater than 90 (Table 4). In particular, general (physical function and psychological state) scores in disabled patients were significantly improved compared with scores of non-disabled patients. This finding contradicts our prior prediction that, due to a lack of cost-effectiveness, the improvement in OHRQoL in disabled patients would be less than in non-disabled patients. It is difficult to determine whether the OHRQoL of disabled or non-disabled individuals can be improved more, but it is obvious that OHRQoL can be significantly improved after fixed implant treatments regardless of disability.

There is a separate classification of severe disabilities only for dental treatment in Korea. It includes severe/mild brain lesions, severe/mild epilepsy, physical disorders above grade 3 (disabilities were classified into 6 grades in Korea before 2019; 1 to 6 corresponds to severe to mild), mental disorder above grade 3, autistic disorder above grade 3, and intellectual disorder above grade 3. This separate classification is set reflecting the difficulties for dental clinicians to treat in 2019 by KMHW. When the severity of disability was categorized in this study, this concept was applied to basic disability classification according to KMHW based on health status. As shown in Table 2, severe disorders affecting treatment (high grade (1–3 grade) mental disability including autism/intellectual disorders, high-grade (1–3 grade) physical disability) were classified as severe disability. Disorders that are not related to oral conditions or low grade (4–6 grade) disability were classified as mild disability. High or low grade mental challenged patients were omitted from the current investigation due to communication limitations. Individuals with brain lesions or epilepsy who express their opinions and physically challenged people who can interact with clinicians were included. As a result, there were no mentally impaired subjects in this OHRQoL trial. Different analysis approaches, such as evaluating lump of food after mastication, may be required for intellectually handicapped patients.

Severely disabled patients in this study have faced major difficulties in obtaining dental treatment due to lack of facilities for general anesthesia or access to specialists. Oral status might be worse among severely disabled patients than mild disabled patients, and treatment may incur significant costs. However, whether satisfaction after dental implant treatments is lesser or greater in severely disabled patients than in mild disabled patients was unknown. In this study, we verified the severity of disabilities that affect improvement of OHRQoL in disabled individuals. Severely disabled people such as individuals with severe brain lesions or other high-grade physical disabilities reported greater satisfaction following implant treatment (*P* = .009). Patients with severe disability experienced difficulties with mastication and swallowing (resulting in digestive problems) and reported dissatisfaction with their lives before implant treatment. Fixed implants seem to effectively address this situation and improve mastication to increase life satisfaction, especially for severe physically disabled patients.

According to the clinical charts of these patients, many of the severely disabled people with partially edentulism did not undergo the requisite number of fixed implant procedures, instead receiving partial restoration in posterior areas for greater function due to financial constraints or anatomical limit. 10 of participants had five posterior implants, 11 received four posterior implants, 31 received three posterior implants, and seven received two posterior implants, and four received only one posterior implant. 32 of them had one to three anterior implants. In any other untreated tooth missing location, there were no removable partial prosthesis. Nevertheless, the improvement of QoL was more significantly increased in severely disabled patients (grade 1–3) than mild disabled patients (grade 4–6) and patients with disabilities irrelevant to oral health (grade 7) (*P* = .030). The null hypothesis was rejected.

When the severity of disability was controlled, we found that a younger age corresponded to better satisfaction with fixed implant treatments. We speculate this is due to a greater willingness to adjust to change in younger patients. With regard to sex, disabled women were previously assumed to show greater satisfaction with dental implant treatments due to a greater desire for esthetics compared with disabled men. However, no difference was found in satisfaction according to sex. People with acquired disabilities were assumed to have higher expectations because they previously had stabilized occlusion and good oral function. However, no difference in satisfaction was found between subjects with congenital and acquired disabilities.

Oral rehabilitation of disabled individuals is closely related to mastication function. Previous studies indicate that disabled individuals have more missing teeth than non-disabled people, which suggests poorer masticatory function of disabled people [3, 4]. Disabled people with a genetic predisposition to rare incurable diseases have poorer masticatory function because of incompatibility with the jaw. Appropriate mastication stimulates the hippocampus in the brain [17, 18]. Thus, without proper occlusion and chewing function improvement, the degree of rehabilitation in patients with brain damage may be insufficient. Dental rehabilitation may be especially beneficial for comprehensive rehabilitation treatment in disabled individuals.

As shown in Table 5, there was no implant failure during the observation period in this study and disabled patients had not more complications with fixed implants than non-disabled patients [19]. However, the mean observation time of 24.71 months after implants was too short for a simple comparison. Long-term studies with long-term results are needed to predict prognosis following this and our previous studies [20]. The success of dental implants is determined by a variety of factors such as surgical technique, bone density, implant design, and the patients’ overall health and oral hygiene care [21]. All of the implants used in this study were internal tapered type and the alveolar bone density for the implants varied. Fortunately, no implants have yet failed; nevertheless, using the osso-densification procedure instead of standard surgical drilling may be more beneficial for disabled patients with poor bone quality [22].

Access to dental care for people with disabilities who rely on caregivers such as nurses, social workers, and family members is generally determined by the amount of time and effort caregivers devote to oral health. However, due to heavy workloads, most caregivers, including professionals, were found to be short on time and to have a poor understanding of dental diseases and their causes [23]. Thus, the importance of oral hygiene and appropriate dental treatments for improving disabled patients’ OHRQoL should be emphasized, and dental personnel should become more involved in the welfare of disabled patients.

This study has limitations due to the small number of subjects and short observation period. More randomized-controlled trials with larger sample sizes and longer observation periods are needed to determine the magnitude of the effects of removable prostheses, such as implant overdentures, as well as fixed implants on OHRQoL in disabled patients. This study examined the OHRQoL of 63 participants after implant treatment, which required them to recall their condition before implant treatment. For more precise results, it should be questioned before and after implant treatment in future research. Furthermore, the number of implants placed each participant was not investigated.

Patients with mental disabilities who were unable to respond to the survey on their own were excluded. This study’s conclusions may be limited because it focuses on physically challenged people. Nonetheless, even for mentally disabled individuals, after dental implants the patients were able to eat better, resulting in being fit according to their caregivers. Obtaining data for a study on those with disabilities is difficult, yet it is vital to increase the welfare of patients. As a result, it is critical to develop OHRQoL assessment methods that may be assessed by caregivers or researchers, such as the Face-scale in circumstances where respondents cannot comprehend the questions or communicate with others.

Implants appear to provide not only oral rehabilitation, but also a new lease on life in disabled patients. Reducing pain and acquiring nutrient-dense food following implants leads to healthier eating habits, physical connection to regular life, and psychological comfort. Many disabled patients, for example, who are unable to lie on the dental chair due to a lack of cooperation, smile at clinicians and appear to try to cooperate with the dental exam after several dental treatments. After receiving dental care, the general health of some patients who were bedridden due to brain injury was greatly improved, allowing them to sit upright in a wheelchair. Therefore, dental treatment for people with disabilities should be improved, and research like this study, despite the limitations, should be carried out.

## Conclusions

Fixed dental implants improved OHRQoL for disabled patients, and the severity of disability was positively correlated with improvement of OHRQoL. For patients with a similar level of disability, the OHRQoL decreased with age. Implant treatment might be challenging for disabled patients due to their unfavorable general health status, lack of cooperation, poor oral hyginene, and financial constraints. But for the welfare of disabled patients, it is necessary to improve OHRQoL for those who have lost their teeth with implant treatment.

## Data Availability

The datasets used and/or analyzed during the current study are available from the corresponding author on reasonable request.

## List of abbreviations

OHRQoL: oral health-related quality of life
OHIP: oral health impact profile
